# Addressing Symptom Burden and Palliative Care Needs in Cystic Fibrosis: A Narrative Review of the Literature

**DOI:** 10.3390/life13081620

**Published:** 2023-07-25

**Authors:** Stephanie DiFiglia, Lara Dhingra, Anna M. Georgiopoulos, Katherine Papia, Erin Sullivan, Amy Plachta, Courtney Boccio, Russell Portenoy, Melissa Basile

**Affiliations:** 1 MJHS Institute for Innovation in Palliative Care, New York, NY 10006, USA; sdifigli@mjhs.org (S.D.); ldhingra@mjhs.org (L.D.); rporteno@mjhs.org (R.P.); 2 Department of Family and Social Medicine, Albert Einstein College of Medicine, Bronx, NY 10461, USA; 3 Department of Psychiatry, Massachusetts General Hospital, Boston, MA 02114, USA; ageorgiopoulos@partners.org; 4 Harvard Medical School, Boston, MA 02115, USA; 5 Cystic Fibrosis Center, Northwell Health, New Hyde Park, NY 11042, USA; khenthorne@northwell.edu (K.P.); esullivan10@northwell.edu (E.S.); cboccio4@gmail.com (C.B.); 6 Cystic Fibrosis Center, Lennox Hill, New York, NY 10021, USA; aplachta1@northwell.edu; 7 Department of Neurology, Albert Einstein College of Medicine, Bronx, NY 10461, USA; 8 Feinstein Institutes for Medical Research, Northwell Health, Manhasset, NY 11030, USA

**Keywords:** cystic fibrosis, symptom burden, palliative care, quality of life

## Abstract

Among people with cystic fibrosis (CF), illness burden is multifaceted, and symptoms may fluctuate in intensity across a lifespan. Caregivers of people with CF may also experience distressing symptoms. Recent developments in CF care, including the availability of highly effective modulator therapies (HEMTs) and new palliative care guidelines promoting palliative care screening may help alleviate symptoms. The objective of this review was to present a narrative view of the recent literature on symptom burden in CF, new screening approaches informed by the Cystic Fibrosis Foundation (CFF) palliative care guidelines, and early data from studies examining the impact of HEMTs on CF symptom burden. A review of the relevant literature was conducted using Google Scholar and PubMed. Included articles covered approaches to burden assessment in CF and other chronic illnesses, epidemiology of CF symptom burden, the impact of HEMTs on symptom burden, and the CFF palliative care guidelines. A primary palliative care model implementing the CFF guidelines was also described. Results of this review show that while recent developments in CF care have led to a reduction in physical symptoms, mental health symptoms remain prevalent. Ongoing screening and triage can ensure that physical symptoms, psychological symptoms, social needs, practical problems, and communication concerns are addressed by care teams.

## 1. Introduction

The illness burden experienced by individuals with cystic fibrosis (CF) and their caregivers can be characterized by problems in many domains (e.g., physical symptoms, psychological distress, and caregiver burden) [1]. The severity of this burden varies across subgroups with CF and may fluctuate across the lifespan and gradually worsen in the context of advanced illness. However, recent advances in novel CF treatments have helped to address symptoms and reduce burden among this population. In particular, the development of highly effective CF transmembrane conductance regulator (CFTR) modulator therapies (HEMTs) that correct the underlying molecular defect causing CF disease [2] has reduced physical symptom severity for many. The promulgation of recent palliative care screening guidelines [3] and recognition of the value of early and ongoing palliative care for CF [4,5] has helped care teams implement new approaches to identify sources of distress among patients and caregivers. Herein, we review the recent literature on the epidemiology of symptom burden in CF, new screening approaches informed by the CF palliative care guidelines, and early data from national studies examining the impact of HEMTs on CF symptom burden.

The objective of this narrative review is to summarize the research literature examining symptom burden in adult and pediatric populations with CF, describe a screening approach informed by the CFF Palliative Care Guidelines, and highlight emerging data on the impact of HEMTs on symptom burden, including a growing body of literature that has focused on family caregivers of CF patients. We also describe the epidemiology of psychological distress among this population. For this narrative review, we included peer-reviewed journal articles and presentations with emerging/preliminary data from 2000–2023. This included research on the prevalence of physical and/or psychological symptoms and distress related to these symptoms and/or associations with symptom distress and other physical or psychological outcomes such as disease severity and quality of life among people with CF and their caregivers. Search terms included “physical symptom burden in cystic fibrosis”, “physical symptom distress cystic fibrosis”, “depression cystic fibrosis”, and “anxiety cystic fibrosis”. Summaries of selected studies can be found in Table 1.

## 2. Symptom Burden and Approaches to Assessment in CF

Symptoms are inherently subjective. In populations with serious chronic illnesses such as CF, there is a very substantial variation in symptom experience and the extent to which one or multiple symptoms is associated with distress. Clinical observations suggest that distress from physical and psychological symptoms is highly prevalent and often undermines quality of life. This is known as symptom burden.

Work supported by the Cystic Fibrosis Foundation (CFF) addressing the quality-of-life concerns of people with CF and their families has long included a focus on symptom management. One important example has been the ongoing effort to develop consensus-based guidelines for pain management [14,15].

In clinical research, symptoms are often considered to be a patient-reported outcome, and studies usually measure symptom burden by the presence of varied symptoms and one or two characteristics, such as intensity or frequency. This measurement approach simplifies a clinical reality that is far more complex. Symptoms are multidimensional phenomena, and clinicians usually can elicit more nuanced information by evaluating each dimension separately [16]. For example, the history can assess *severity* (distinguishing intensity right now, on average, and at its worst); *temporal features* (describing rapidity of onset, course over time, overall duration, and episodic components); *quality* (pain, for example, may be aching, sharp, dull, or burning); *bodily areas affected*; and *factors that worsen or ameliorate the symptom experience* (Table 2).

Relatedly, each symptom can be characterized by the extent to which it adversely affects physical functioning, mood, and other aspects of psychological well-being, role functioning, and relationships with others, as well as overall quality of life. Symptoms also may be characterized by the meaning attributed to them; patients may describe a symptom in terms of organ damage, worsening illness, need for a change in therapy, or in terms of its impact on family burden.

This abundant clinical information about the symptoms experienced by people with CF often influences management approaches. Symptom assessment may guide the selection of treatments for symptom-focused therapy and may also illuminate an etiology or pathophysiology for the symptom that could be targeted for intervention.

## 3. Epidemiology of Symptom Burden in Community-Dwelling Populations with CF

The high prevalence and multidimensional nature of symptoms has been reported by numerous studies. Most of these studies have been conducted in seriously ill populations with cancer or other illnesses [18,19,20,21,22,23]. Although relatively few studies have evaluated the heterogeneous population with CF, the extant literature suggests that CF is like other chronic illnesses in terms of the burden and distress associated with numerous common symptoms [11,24]. These data suggest that adults with CF are more likely to report physical symptom burden than children or adolescents [4,6] and that women and older people have relatively higher symptom prevalence and severity [1,4,6]. The most prevalent physical symptoms are cough, shortness of breath, fatigue, sinus discharge, difficulty sleeping, and pain [1,5,11,12]. Cough, shortness of breath, sinus discharge, and fatigue often are rated as the most severe or distressing symptoms [1,5,11,25]. Additionally, initial data from one trial in 61 adults with CF found that a significant portion of participants reported cough and fatigue as highly distressing (i.e., “quite a bit” or “very much” distressing) at baseline, as well as at 3-, 6-, and 9-month follow-up assessments [26]. This study also showed that mean distress scores for physical symptoms commonly rated as highly distressing (i.e., fatigue, dyspnea, cough, sleep disturbance, and drowsiness) remained highly distressing over nine months [26]. Additionally, some symptoms with lower prevalence rates have been reported as severe or highly distressing (e.g., gastrointestinal symptoms) to patients [5,11].

Physical symptom burden is important to assess given its strong association with health-related quality of life (HRQOL). For example, one study with 87 children and adolescents receiving care at CF centers showed that sleep disturbance was associated with lower HRQOL scores [27]. Several studies of adolescents and adults treated in CF centers show that higher symptom burden and the negative effects of physical symptoms on function may also associate with worse pulmonary function (lower FEV1% predicted), which can lead to poorer HRQOL [1,27,28,29].

Psychological symptoms also are common and burdensome in the population with CF. Indeed, a recent investigation of 256 adults across five U.S. CF centers who completed the Integrated Palliative Care Outcome Scale [30] observed that feeling depressed and anxious were more frequent sources of illness burden than most physical symptoms [4,31] suggesting the potentially larger impact of psychological symptoms on burden in the era of highly effective modulator therapy.

People with CF report higher levels of depression and anxiety than the general population [5,7,8,9,10,13]. In a large study of 6088 people with CF aged 12 and older across nine countries, 11% had mild to severe depression scores on the Hospital Anxiety and Depression Scale (HADS-D [32]; range 8–15%), and 27% had elevated depression scores on the Center for Epidemiologic Studies-Depression Scale (CES-D [33]; range 20–31%). An average of 30% endorsed mild to severe anxiety, as measured on the HADS-Anxiety subscale [34]. Other studies show that feeling irritable, sad, or worried—which are often symptoms of depression or anxiety—are among the most distressing symptoms reported among adults with CF [1,5,11]. Initial data from one longitudinal study examining the trajectory of symptoms in 61 adults with CF found that symptoms rated as highly distressing (i.e., “quite a bit” or “very much” distressing) by a significant portion of participants included irritability, sadness, and concentration problems. While these symptoms became less distressing for some adults over the course of nine months, they still remained highly distressing for a portion of adults [26]. This suggests that symptoms of depression and anxiety are commonly experienced by people with CF, and CF care teams can consider addressing the range of problems summarized in Table 3.

Depressive and anxiety symptoms have been found to associate with gastrointestinal and respiratory symptom distress [1] and with some measures of more severe disease severity, such as increased pulmonary exacerbations [36,37], poorer lung function [36,38], increased healthcare utilization [39], and increased mortality [13]. Other measures of disease severity, including lower FEV1% predicted and lower body mass index, are not significantly associated with psychological distress [1,11].

In a recent qualitative study of 16 adults managed by three U.S. CF centers, psychological and physical symptoms were found to interact [40]. Specifically, patients perceived that poor mood undermined their treatment adherence, CF-related exacerbations initiated a sense of loss of control, and increased severity of one type of symptom (physical or psychological) compounded the burden of the other [40]. Other recent qualitative studies have identified psychological burden among specific subgroups of people with CF, including those with CF-related diabetes, those taking a HEMT, and those ineligible for modulator therapy. For example, people with CF-related diabetes reported experiencing negative feelings about the diagnosis of diabetes, such as difficulty coping and shock, and increased treatment burden from managing two chronic conditions [41]. People with CF who are ineligible for HEMT have reported feelings of sadness, disappointment, and hopelessness about ineligibility. Although those taking HEMT may experience lower psychological burden, they commonly report ongoing anxiety, fear, and grief [42].

Psychological distress is highly prevalent among family caregivers of people with CF, who also have higher rates of depression and anxiety compared to the general population [7,8,9,10]. Quittner and colleagues [34] found that 4102 caregivers of young children with CF often showed similar or higher levels of depressive symptomatology than adolescents and adults with CF. Additionally, they observed that anxiety symptoms were present in nearly half of mothers on average (48%) and over one-third of fathers (36%) across eight European countries and the U.S. [34].

In summary, epidemiologic findings highlight the need to assess both physical and psychological symptoms in both adults and children with CF and the family who are caregivers. Assessing symptoms as part of a broader evaluation of quality of life is a palliative care best practice and should be performed repeatedly to capture changes that occur over time [43].

## 4. The Impact of HEMTs on Symptom Burden in CF

Recent developments in CF treatments include the widespread availability of HEMTs—particularly the triple combination drug elexacaftor/tezacaftor/ivacaftor [44,45]—which may reduce symptom burden among people with CF. An unpublished cross-sectional pilot study (AHRQ R03HS026970) [46] that used the Cystic Fibrosis Questionnaire-Revised [47] (CFQ-R) to assess the impact of treatment adherence on quality of life among 28 adults with CF currently prescribed an HEMT found associations among the use of HEMTs, reduced symptom burden, and higher quality of life. The study found that while mean quality of life scores were moderate to high across all domains, mean scores were typically higher (i.e., better) among those domains associated with physical symptom burden, such as physical functioning, eating, respiratory symptoms and digestion as compared to those domains associated with psychological symptoms, perceived treatment burden, and perceived overall health. These initial findings could suggest that while HEMTs have led to decreased physical symptom burden, psychological and other non-physical symptoms continue to be a source of distress in the CF population.

The following description reflects the experience of symptom burden before and after starting the HEMT Trikafta from one individual with CF:

“Life before Trikafta and life after Trikafta. This surreal, mutually understood concept is one that I’ve often seen discussed amongst members of the CF community. Trikafta came into my life when I was at the lowest point that I had ever experienced. In 2017, I fell into a cycle of aggressive lung infections and sickness that I could not shake. For the first time, I experienced the fear of realizing that I had developed resistance to the IV antibiotic regimens and treatments that had worked for my lungs countless times before. I was scared and felt hopeless and I was unsure if my body could actually pull itself out of this worsening spiral. Every day I felt weak and tired and felt the very palpable effects of my failing health. Managing both the physical and mental symptoms of my progressing disease was overwhelming. I wondered after living a seemingly “normal”, active life filled with very little restrictions, if this was the chain of events that would negatively change the course of the rest of my life. I essentially had to put my entire life and future on hold and spent every day fighting to try to nurse my body and lungs back to health, spending hours daily on treatments and searching for something to pull me out of the trenches. After about a year of fighting through a time that felt like a terrible dream, I emerged as a different person. My baseline lung function had decreased approximately 15–20%, I lost a noticeable amount of weight, and I felt like a shell of my younger and healthier self both mentally and physically. For the first time in my life, I actually looked ill. I was unsure if the future I had always pictured myself building without question would even be possible or if I would be able to pursue my dream of working in medicine. Then my own personal miracle happened. In 2018, I was given the opportunity to be a phase 3 clinical trial participant for Trikafta- and the rest is history.

Five years later, life after Trikafta is a beautiful life that frankly at one point I did not think was in the cards for me. Within 24 h of my first dose, I entered a transformation period where my entire body started to change. Essentially every symptom that I experienced my entire life began to slowly disappear, including being freed from my classic chronic CF cough. I am healthier now than I have ever been. I am a version of myself far beyond what I could have ever imagined was possible for my body to achieve. I have been able to phase out almost all of my previous CF treatments and I have had no indication for IV antibiotics, sinus surgery, or hospitalization, when pre-Trikafta I would undergo these types of therapies every 6–12 months. I learned what it feels like to truly be able to breathe. I am active and can run, I can maintain a healthy body weight, my mental health and quality of life has vastly improved, and most significantly, I experienced approximately a 20% increase in FEV1 lung function. As I approach my 30th birthday I see a future filled with opportunity. I am preparing to graduate from graduate school and enter the field of medicine as a medical provider- a full circle milestone. Watching my body and life transform over these last five years has filled me with such gratitude for medical research. Trikafta is changing the face of CF and giving patients the opportunity to be the authors of their own futures, just like it did for me. I am eternally grateful to have been saved by science”.

It should be noted that this piece reflects the perspective of one person with CF currently taking the HEMT Trikafta and may not reflect the experiences of all people with CF, particularly those not taking this HEMT.

## 5. Palliative Care in CF and the CFF Palliative Care Guidelines

Palliative care is a clinical approach provided by an interdisciplinary team to decrease symptom burden and improve the quality of life for patients with serious chronic illness and their families [42,48]. However, until recently, there were no recommendations to inform CF-specific palliative care. To address this gap, in 2017, the Cystic Fibrosis Foundation convened a committee to develop consensus-based guidelines for delivering palliative care to people with CF and their family members [3]. This included defining palliative care for CF. Using a modified Delphi methodology, the members developed the following definition: “Palliative care focuses on reducing physical and emotional symptoms and improving quality of life for people with CF throughout their lives. Palliative care occurs alongside usual treatments and is individualized according to the unique goals, hopes and values of each person with CF” [49]. The guidelines offer recommendations to CF care teams for repeated screening throughout the illness course for a range of unmet needs, including physical and psychological symptoms, communication issues, and practical concerns for people with CF, and also screening for burden among family caregivers.

The recommendations specify using the Integrated Palliative Care Outcome Scale [30] to screen for unmet needs among people with CF 12 years and older, the Brief Assessment Scale for Caregivers (BASC [50]) to assess caregiver needs and burden, and additional optional measures to further clarify identified concerns. The latter include the Patient Health Questionnaire-9 item Scale (PHQ-9 [51]) and the Generalized Anxiety Disorder-7 item Scale (GAD-7 [52]) to measure depression and anxiety, respectively, among people with CF and caregivers. Other optional measures for individuals with CF include the Spiritual Needs Assessment for Patients (SNAP [53]) to assess the desire for help with unmet psychosocial, spiritual/existential, and religious needs, and one or more of the Cystic Fibrosis Questionnaire-Revised (CFQ-R [54]), subscales to assess HRQOL. An additional measure for caregivers is the Prolonged Grief Disorder (PG-13 [55]), which may be given to caregivers following the death of a loved one with CF, to screen for persistent complex bereavement disorder. For children with CF under age 12, the guidelines recommend using the Integrated Palliative Care Outcome Scale to guide conversations with children and their caregivers to identify unmet palliative care needs [3]. In summary, the CFF consensus guidelines define palliative care for CF and provide direction for CF care teams to integrate primary palliative care into their standard clinic care, with recommendations for referral to specialty palliative care when necessary.

*The Improving Life with CF pragmatic implementation trial*: Routine screening for unmet palliative care needs with subsequent triage by the CF care team (i.e., *primary palliative care*) may be one approach for addressing symptoms and distress throughout the life course of people with CF and their families. To implement and test such an approach, the *Improving Life with CF* study, a pragmatic implementation trial of a multicomponent primary palliative care intervention, is currently underway in five U.S. CF centers (Massachusetts General Hospital, Northwell Health North Shore, Northwell Health Lenox Hill, Stony Brook University, and Emory University; Principal Investigators: Anna Georgiopoulos and Lara Dhingra). The intervention model comprised (1) screening-and-triage care processes, (2) CF provider education, featuring a 6 h Train-the-Trainer curriculum and 11 accredited webinars, (3) seven best-practice treatment guides for addressing high frequency symptoms, (4) 10 patient educational handouts for self-managing common symptoms, and (5) a quality improvement toolkit; the model includes both foundational elements concordant with the CFF Models of Palliative Care Delivery guidelines [3] (e.g., routine screening for unmet needs in multiple domains using the IPOS [30] and the BASC [50,56]) and modifiable, CF center-specific elements (e.g., methods and processes for screening patients/caregivers can be customized based on available resources or patient/caregiver preference). As part of the model’s national implementation trial, the “Assessing for Distressing Symptoms And Palliative Care Needs through Targeted Communication” (ADAPT-CF) guide has also been created by interdisciplinary pediatric, palliative care, and communication experts and piloted to assess illness burden in children with CF under age 12 [57]. The primary outcome analysis in this ongoing trial will evaluate the change in illness burden on the IPOS between baseline and the outcome assessment following 22 months of the intervention’s implementation at each site (www.improvinglifewithcf.org (accessed on 1 May 2023)) [58].

## 6. Discussion

In this review, we have described the nature of symptom burden in the care of patients with a serious chronic illness like CF, reviewed state-of-the-art approaches for symptom assessment, discussed the epidemiology of symptoms in the CF population and the recent widespread use of HEMTs, as well as recent developments to inform CF care, including the CFF palliative care guidelines and a large ongoing national implementation trial. This latter work supports the need for ongoing screening-and-triage in CF centers to ensure that psychological symptoms, social needs, practical problems, and communication concerns are also addressed by care teams in addition to physical symptoms.

Prior studies highlighted that symptom distress may vary widely based on a patient’s age, disease severity, and many other characteristics, including the current use of treatments. HEMTs are altering the prevalence and severity of CF symptoms [59], particularly among younger patients who have initiated HEMTs at earlier ages. For example, physical symptoms may be less severe in this latter group. By offering regular multidomain screening that is not solely limited to physical symptoms, but detects other types of problems and clinical concerns, CF providers may identify sources of distress and offer effective, tailored interventions to address these issues and possibly improve quality of life.

At the present time, not all individuals living with CF are eligible for the use of HEMTs, as these therapies are not effective for altering the effects of all CF genetic variants [45]. Furthermore, there are no data on the long-term effects of HEMTs, and it is not known if symptoms may re-emerge over time. Efforts are needed to assess the long-term impact of routine palliative care and HEMT use on HRQOL and symptom burden and to determine the impact on CF-related treatment outcomes.

For example, studies have begun to explore the impact of reducing complex medication regimens as a means of alleviating treatment burden in CF [60]. Recently, the CF medication–withdrawal study SIMPLIFY-IP-19 (“study to evaluate stopping inhaled hypertonic saline or dornase alfa in people with CF who are taking Trikafta”) looked at the effects of discontinuing hypertonic saline and pulmozyme while using HEMT [60]. Early results from this open-label study in 477 adolescents and adults randomly assigned to either continue or discontinue hypertonic saline showed that the discontinuation of the drugs did not have a meaningful effect on lung function at 6 weeks; in other words, it did not result in a reduction in lung function, suggesting that there is potential to reduce medication burden in CF patients taking HEMT. However, as this was not a long-term study, a longitudinal analysis of respiratory symptoms and lung function among a larger multi-age sample is ongoing.

Another area that warrants additional study is the long-term impact of HEMTs on mental health and coping. Further research is needed to better understand the complex connections between mental health and modulators, both for those using HEMT and those who are intolerant or ineligible, as well as how optimal delivery of CF primary palliative care is evolving in the modulator era [61,62,63,64,65,66].

## 7. Conclusions

While HEMTs may reduce physical symptom burden, their long-term impact on CF symptoms remains unknown, and CF continues to profoundly affect the lives of many people with CF and their families. Multidisciplinary CF teams can help to improve quality of life by systematically identifying unmet needs in multiple domains—physical, psychological, communication needs, spiritual/existential distress, and practical problems—and delivering primary palliative care interventions.

## Figures and Tables

**Table 1:** Summary of Selected Studies on CF Symptom Burden and Palliative Care Needs *.

Reference	Study Design	Sample	Outcome
Sawicki et al., 2008 [1]	Prospective longitudinal	303 adults with CF	Results show the presence of physical symptoms: respiratory (cough and shortness of breath), fatigue, and sleep disturbances and psychological symptoms: worrying, feeling irritable, and feeling sad. The most distressing symptoms were fatigue and irritability.
Stenekes et al., 2009 [6]	Cross-sectional	123 adults and children with CF	A total of 84% of participants reported pain (with headache and abdominal pain reported most frequently). A total of 83% reported cough, with 63% of participants with cough reporting that cough always or sometimes interfered with sleep. A total of 64% of participants reported breathlessness.
Goldbeck et al., 2010 [7]	Case-control study	670 German adolescents and adults with CF	CF patients showed high levels of anxiety (20.6%) and depression (9.6%). Adults with CF had more elevated anxiety levels than healthy controls.
Besier and Goldbeck, 2011 [8]	Dyadic-based survey	162 German adolescents with CF and associated parental caregivers	Caregivers reported significantly increased rates of symptoms of anxiety and depression (38.0%) compared to adolescent CF patients (8.0–12.0%).
Ploessl et al., 2014 [9]	Retrospective chart review	190 children with CF	A total of 9% of CF patients had a diagnosis of depression. CF patients with depression had lower FEV1% predicted, more hospitalizations, and more CF exacerbations requiring treatment each year than patients without depression.
Quittner et al., 2014 [10]	Cross-sectional	6088 adolescents and adults with CF and 4102 parents of children with CF from Europe and the U.S.	Overall, anxiety and depression symptoms were elevated in patients with CF and parents at a rate two to three times that of non-CF community samples.
Friedman et al., 2018 [11]	Prospective longitudinal (Note: article only reports on data from enrollment and baseline surveys)	41 adults and adolescents with CF	At baseline, patients reported numerous physical (cough, lack of energy, shortness of breath, pain, difficulty sleeping) and psychological symptoms (feeling sad, worrying, feeling nervous). Many psychological symptoms were rated as more distressing than physical symptoms. Anxiety and depression correlated with physical symptom distress and difficulty with CF self-management, but not with disease severity.
Dhingra et al., 2020 [5]	Prospective longitudinal	74 adults with CF	Participants completed a monthly online screening, in which two consecutive screenings revealing one or more “indicators of concern” triggered an attempted triage with follow-up by CF professionals. In total, 164 attempted triages resulted in 84 completed triages (51.2%), of which 39 (46.4%) required prompt follow-up.
Trandel et al., 2020 [12]	Cross-sectional	164 adults with CF	A total of 78% of participants reported ≥1 unmet palliative care need. Needs relating to physical and daily living (72%) and psychological domains (66%) were the most prevalent.
Schechter et al., 2021 [13]	Multi-center observational	1005 adolescents and adults with CF across nine countries	A total of 25.1% of participants had a positive screen for depression, and 33.6% had a positive screen for anxiety. A positive screening result for depression was associated with an increased risk of 5-year mortality among adults with CF.

* Ordered by year.

**Table 2:** Approach to symptom assessment [17].

**Symptom Characteristics**	
**Severity**	Numeric scale (“0–10” where “0” is not intense at all, and “10” is the highest intensity; distinguishes “symptom severity right now”, “symptom severity on average during the past week”, and “worst severity during the past week”)
	Verbal rating scale (“none”, “moderate”, “severe”; distinguish “symptom severity right now”, symptom severity on average during the past week”, and “worst severity during the past week”)
**Temporal features**	Onset (rapid or gradual)
	Duration
	Course stable, improving, worsening, or fluctuating
	Presence of episodic flares
**Location**	Focal, multifocal, or generalized
	Referral pattern
**Quality**	Examples for pain include “aching”, “throbbing”, or “burning”
**Exacerbating or relieving factors**	Examples include coughing, eating, specific positions
**Essential Historical Information**	
Premorbid and comorbid medical and psychiatric conditions	
Prior evaluation for the symptom	
Prior treatments	

Reprinted with permission from www.improvinglifewithcf.org (accessed on 1 May 2023).

**Table 3:** Symptoms to consider in the assessment and management of anxiety and depression [35].

**1. Core depression symptoms**
Sad, depressed, or irritable mood
Lack of interest or pleasure (e.g., social withdrawal, disengagement from usual activities)
**2. Additional depression symptoms**
Trouble sleeping or sleeping too much
Decreased or increased appetite, weight change
Impaired concentration, low motivation, or decline in academic/work performance
Fatigue, low energy
Feelings of guilt, worthlessness, or being a burden on others
Low libido
Increased sensitivity to criticism or perceived rejection
Trouble making decisions, feeling helpless
Hopelessness, loss of meaning, emptiness
Thoughts of death or suicide
**3. Core anxiety symptoms**
Anxious, worried, fearful, or irritable mood
Avoidance of anxiety-provoking stimuli
**4. Additional anxiety/traumatic stress symptoms**
**Generalized anxiety**: Excessive, uncontrollable worry in multiple situations Trouble sleeping, fatigue Impaired concentration Muscle tension, restlessness Other physical symptoms: headache, abdominal pain, nausea, diarrhea
**Panic attack**: Intense fear with abrupt onset Tachycardia, palpitations, chest pain Dyspnea, choking sensation Sweating, shaking, paresthesia, feeling dizzy, hot, or cold GI distress, nausea Feelings of unreality, fear of losing control or dying
**Social anxiety**: Excessive fear or panic in social situations Fear of rejection, negative evaluation, humiliation Reluctance to speak in public or to peers Avoidance of social situations
**Specific phobia**: Excessive fear, panic, or distress related to a specific object or situation Typical examples include animals (snakes), heights, enclosed spaces In people with CF, some phobias may be better characterized as sequelae of medical traumatic stress (e.g., intense fears of medical settings or procedures, needles, hypoglycemia, hemoptysis)
**Separation anxiety**: Persistent anxiety with separation, excessive for developmental stage (e.g., in children or adolescents) High distress when anticipating or during separation from attachment figure Worry something bad will happen to loved ones Refusal to leave home or to be alone (e.g., in part of the house, when sleeping)
**Traumatic stress/Post-traumatic stress disorder (PTSD)**: Follows discrete or chronic traumatic stressor(s) Intrusion—nightmares, flashbacks, distressing memories Avoidance of related stimuli Change in thoughts and feelings (e.g., feeling hopeless, detached, or emotionally numb)—can mimic depression or anxiety Hyperarousal or reactivity (poor sleep, poor concentration, increased startle, hypervigilance) Watch for physical symptoms, impaired self-regulation, risky behavior, or trouble maintaining relationships
**Obsessive compulsive disorder (OCD)**: A pattern of specific intrusive thoughts causing anxiety (i.e., obsessions) and/or rituals aimed at reducing anxiety (i.e., compulsions) Obsessions about germs, harm to others, order, unwanted religious or sexual thoughts Rituals, such as excessive washing hands, counting, reciting, seeking excessive reassurance, checking, maintaining specific order
**5. Emergent and urgent symptoms and signs**
**Requiring immediate attention** Wish to be dead, thoughts of self-harm or suicide, self-injurious behavior Extreme irritability or anger; reckless behavior; aggression Minimal oral intake, severe weight loss Rapidly fluctuating mental status or autonomic changes—consider delirium, medication side effect, substance intoxication or withdrawal, serotonin syndrome Psychotic symptoms (delusions, hallucinations) or catatonia (a state of abnormal behavior and movement resulting in stillness or extreme restlessness)
**Requiring further evaluation** Rapid worsening from baseline Psychomotor slowing (slow, quiet speech, limited activity or movement) Psychomotor agitation (pacing, restlessness) Poor self-care in daily activities or for medical needs School refusal (children) or missing work (adults) Comorbid alcohol or substance misuse Rapidly shifting or elevated/irritable mood, increased energy, impulsivity—consider bipolar disorder, another disorder of emotion regulation (borderline personality disorder or other personality disorders), medication side effect, substance intoxication or withdrawal

Reprinted with permission from www.improvinglifewithcf.org (accessed on 1 May 2023).

## Data Availability

Not applicable.

## References

[1] Sawicki G.S., Sellers D.E., Robinson W.M. (2008). Self-reported physical and psychological symptom burden in adults with cystic fibrosis. J. Pain Symptom Manag..

[2] Caverly L.J., Riquelme S.A., Hisert K.B. (2022). The Impact of Highly Effective Modulator Therapy on Cystic Fibrosis Microbiology and Inflammation. Clin. Chest Med..

[3] Kavalieratos D., Georgiopoulos A., Dhingra L., Basile M., Rabinowitz E., Hempstead S., Faro A., Dellon E.P. (2020). Models of palliative care delivery for individuals with cystic fibrosis: Cystic Fibrosis Foundation evidence-informed consensus guidelines. J. Palliat. Med..

[4] Di Figlia S., Georgiopoulos A., Portenoy R., Berdella M., Friedman D., Kier C., Linnemann R., Middour-Oxler B., Walker P., Wang J. (2022). Palliative care needs in cystic fibrosis (CF): Baseline data from the Improving Life with CF multi-site implementation trial for primary palliative care intervention. J. Cyst. Fibros..

[5] Dhingra L., Walker P., Berdella M., Plachta A., Chen J., Fresenius A., Balzano J., Barrett M., Bookbinder M., Wilder K. (2020). Addressing the burden of illness in adults with cystic fibrosis with screening and triage: An early intervention model of palliative care. J. Cyst. Fibros..

[6] Stenekes S.J., Hughes A., Grégoire M.C., Frager G., Robinson W.M., McGrath P.J. (2009). Frequency and self-management of pain, dyspnea, and cough in cystic fibrosis. J. Pain Symptom Manag..

[7] Goldbeck L., Besier T., Hinz A., Singer S., Quittner A.L. (2010). Prevalence of symptoms of anxiety and depression in German patients with cystic fibrosis. Chest.

[8] Besier T., Goldbeck L. (2011). Anxiety and depression in adolescents with CF and their caregivers. J. Cyst. Fibros..

[9] Ploessl C., Pettit R.S., Donaldson J. (2014). Prevalence of depression and antidepressant therapy use in a pediatric cystic fibrosis population. Ann. Pharmacother..

[10] Quittner A.L., Goldbeck L., Abbott J., Duff A., Lambrecht P., Solé A., Tibosch M.M., Bergsten Brucefors A., Yüksel H., Catastini P. (2014). Prevalence of depression and anxiety in patients with cystic fibrosis and parent caregivers: Results of The International Depression Epidemiological Study across nine countries. Thorax.

[11] Friedman D., Linnemann R.W., Altstein L.L., Islam S., Bach K.T., Lamb C., Volpe J., Doolittle C., St John A., O’Malley P.J. (2018). The CF-CARES primary palliative care model: A CF-specific structured assessment of symptoms, distress, and coping. J. Cyst. Fibros..

[12] Trandel E.T., Pilewski J.M., Dellon E.P., Jeong K., Yabes J.G., Moreines L.T., Arnold R.M., Hoydich Z.P., Kavalieratos D. (2020). Prevalence of unmet palliative care needs in adults with cystic fibrosis. J. Cyst. Fibros..

[13] Schechter M.S., Ostrenga J.S., Fink A.K., Barker D.H., Sawicki G.S., Quittner A.L. (2021). Decreased survival in cystic fibrosis patients with a positive screen for depression. J. Cyst. Fibros..

[14] Havermans T., Colpaert K., De Boeck K., Dupont L., Abbott J. (2013). Pain in CF: Review of the literature. J. Cyst. Fibros..

[15] Dhingra L.D.E., Georgiopoulos A., Portenoy R.K. Supporting individuals with cystic fibrosis and their families across the lifespan: Serious illness discussions. Proceedings of the Navigating Healthcare & Psychosocial Transitions through the Lifespan, Proceedings of the North American Cystic Fibrosis Conference.

[16] Chang V.T., Bruera E., Higginson I.J., von Gunten C.F., Morita T. (2021). Tools for Pain and Symptom Asssessmen. Textbook of Palliative Medicine and Supportive Care.

[17] Portenoy R.K. (2021). Best Practice Guide: Assessment and Management of Pain. Improving Life with CF: A Primary Palliative Care Partnership (Cystic Fibrosis Foundation, GEORGI19QI0). https://www.improvinglifewithcf.org/wp-content/uploads/2021/01/BPG-and-ADAPT-CF-PDFs.zip.

[18] Crawley L., Payne R., Bolden J., Payne T., Washington P., Williams S. (2000). Palliative and end-of-life care in the African American community. JAMA.

[19] Eckerblad J., Theander K., Ekdahl A.W., Jaarsma T. (2016). Symptom trajectory and symptom burden in older people with multimorbidity, secondary outcome from the RCT AGe-FIT study. J. Adv. Nurs..

[20] Kirkova J., Walsh D., Rybicki L., Davis M.P., Aktas A., Tao J., Homsi J. (2010). Symptom severity and distress in advanced cancer. Palliat. Med..

[21] Solano J.P., Gomes B., Higginson I.J. (2006). A comparison of symptom prevalence in far advanced cancer, AIDS, heart disease, chronic obstructive pulmonary disease and renal disease. J. Pain Symptom Manag..

[22] Walke L.M., Gallo W.T., Tinetti M.E., Fried T.R. (2004). The burden of symptoms among community-dwelling older persons with advanced chronic disease. Arch. Intern. Med..

[23] Zimmermann C., Riechelmann R., Krzyzanowska M., Rodin G., Tannock I. (2008). Effectiveness of specialized palliative care: A systematic review. JAMA.

[24] Dhingra L.K., Lam K., Cheung W., Shao T., Li Z., Van de Maele S., Chang V.T., Chen J., Ye H., Wong R. (2015). Variation in symptom distress in underserved Chinese American cancer patients. Cancer.

[25] Basile M.J., Dhingra L., DiFiglia S., Polo J., Portenoy R., Wang J., Walker P., Middour-Oxler B., Linnemann R.W., Kier C. (2023). Development of a Cystic Fibrosis Primary Palliative Care Intervention: Qualitative Analysis of Patient and Family Caregiver Preferences. J. Patient Exp..

[26] Walker P.B.M., Plachta A., Wilder K., Chen J., Schwind E., Glajchen M., Bookbinder M., Portenoy R., Dhingra L. An early intervention palliative care model for cystic fibrosis patients. Proceedings of the Oral Presentation at the Cystic Fibrosis Foundation Palliative Care Workshop.

[27] Dill E.J., Dawson R., Sellers D.E., Robinson W.M., Sawicki G.S. (2013). Longitudinal trends in health-related quality of life in adults with cystic fibrosis. Chest.

[28] Gee L., Abbott J., Conway S.P., Etherington C., Webb A.K. (2003). Quality of life in cystic fibrosis: The impact of gender, general health perceptions and disease severity. J. Cyst. Fibros..

[29] Habib A.R., Manji J., Wilcox P.G., Javer A.R., Buxton J.A., Quon B.S. (2015). A systematic review of factors associated with health-related quality of life in adolescents and adults with cystic fibrosis. Ann. Am. Thorac. Soc..

[30] Murtagh F.E., Ramsenthaler C., Firth A., Groeneveld E.I., Lovell N., Simon S.T., Denzel J., Guo P., Bernhardt F., Schildmann E. (2019). A brief, patient- and proxy-reported outcome measure in advanced illness: Validity, reliability and responsiveness of the Integrated Palliative care Outcome Scale (IPOS). Palliat. Med..

[31] Cleeland C.S. (2007). Symptom Burden: Multiple Symptoms and Their Impact as Patient-Reported Outcomes. JNCI Monogr..

[32] Zigmond A.S., Snaith R.P. (1983). The hospital anxiety and depression scale. Acta Psychiatr. Scand..

[33] Radloff L.S. (1991). The use of the Center for Epidemiologic Studies Depression Scale in adolescents and young adults. J. Youth Adolesc..

[34] Quittner A.L., Zhang J., Marynchenko M., Chopra P.A., Signorovitch J., Yushkina Y., Riekert K.A. (2014). Pulmonary medication adherence and health-care use in cystic fibrosis. Chest.

[35] Georgiopoulos A.M., Friedman D., Sher Y. (2021). Best Practice Guide: Management of Depression and Anxiety. Improving Life with CF: A Primary Palliative Care Partnership (Cystic Fibrosis Foundation, GEORGI19QI0). https://www.improvinglifewithcf.org/wp-content/uploads/2021/01/BPG-and-ADAPT-CF-PDFs.zip.

[36] Riekert K.A., Bartlett S.J., Boyle M.P., Krishnan J.A., Rand C.S. (2007). The Association Between Depression, Lung Function, and Health-Related Quality of Life Among Adults With Cystic Fibrosis. Chest.

[37] Yohannes A.M., Willgoss T.G., Fatoye F.A., Dip M.D., Webb K. (2012). Relationship between anxiety, depression, and quality of life in adult patients with cystic fibrosis. Respir. Care.

[38] Fidika A., Herle M., Goldbeck L. (2014). Symptoms of depression impact the course of lung function in adolescents and adults with cystic fibrosis. BMC Pulm. Med..

[39] Snell C., Fernandes S., Bujoreanu I.S., Garcia G. (2014). Depression, illness severity, and healthcare utilization in cystic fibrosis. Pediatr. Pulmonol..

[40] Friedman D., Kaskas M.M., Quittner A.L., Smith B.A., Georgiopoulos A.M. (2022). Patient engagement in the development of CF-CBT: A cystic fibrosis-specific cognitive-behavioral intervention for adults. Front. Psychol..

[41] Collins S., Jones A., Woodward S., Sturt J. (2022). The experience of living with and managing cystic fibrosis related diabetes: A qualitative review. J. Res. Nurs..

[42] Keyte R., Kauser S., Mantzios M., Egan H. (2022). The psychological implications and health risks of cystic fibrosis pre- and post- CFTR modulator therapy. Chronic Illn..

[43] Vandeleur M., Walter L.M., Armstrong D.S., Robinson P., Nixon G.M., Horne R.S.C. (2017). How Well Do Children with Cystic Fibrosis Sleep? An Actigraphic and Questionnaire-Based Study. J. Pediatr..

[44] Zaher A., ElSaygh J., Elsori D., ElSaygh H., Sanni A. (2021). A Review of Trikafta: Triple Cystic Fibrosis Transmembrane Conductance Regulator (CFTR) Modulator Therapy. Cureus.

[45] Nichols D.P., Paynter A.C., Heltshe S.L., Donaldson S.H., Frederick C.A., Freedman S.D., Gelfond D., Hoffman L.R., Kelly A., Narkewicz M.R. (2022). Clinical Effectiveness of Elexacaftor/Tezacaftor/Ivacaftor in People with Cystic Fibrosis: A Clinical Trial. Am. J. Respir. Crit. Care Med..

[46] Basile M.W.J., Davdison K., Pullen E., Perry B., Hajizadeh N., Zhang M. Factors for Poor Adherence among Adults with Cystic Fibrosis. R03HS026970 Agency for Healthcare Research and Quality (AHRQ): 2020–2022. https://grantome.com/grant/NIH/R03-HS026970-02.

[47] Quittner A.L., Buu A., Messer M.A., Modi A.C., Watrous M. (2005). Development and validation of The Cystic Fibrosis Questionnaire in the United States: A health-related quality-of-life measure for cystic fibrosis. Chest.

[48] Morrison R.S., Meier D.E. (2004). Clinical practice. Palliative care. N. Engl. J. Med..

[49] Dellon E.P., Goggin J., Chen E., Sabadosa K., Hempstead S.E., Faro A., Homa K. (2018). Defining palliative care in cystic fibrosis: A Delphi study. J. Cyst. Fibros..

[50] Glajchen M., Kornblith A., Homel P., Fraidin L., Mauskop A., Portenoy R.K. (2005). Development of a brief assessment scale for caregivers of the medically ill. J. Pain Symptom Manag..

[51] Kroenke K., Spitzer R.L., Williams J.B. (2001). The PHQ-9: Validity of a brief depression severity measure. J. Gen. Intern. Med..

[52] Spitzer R.L., Kroenke K., Williams J.B., Löwe B. (2006). A brief measure for assessing generalized anxiety disorder: The GAD-7. Arch. Intern. Med..

[53] Sharma R.K., Astrow A.B., Texeira K., Sulmasy D.P. (2012). The Spiritual Needs Assessment for Patients (SNAP): Development and validation of a comprehensive instrument to assess unmet spiritual needs. J. Pain Symptom Manag..

[54] Modi A.C., Quittner A.L. (2003). Validation of a disease-specific measure of health-related quality of life for children with cystic fibrosis. J. Pediatr. Psychol..

[55] Tsai W.I., Kuo S.C., Wen F.H., Prigerson H.G., Tang S.T. (2018). Prolonged grief disorder and depression are distinct for caregivers across their first bereavement year. Psycho-Oncology.

[56] Wojtaszczyk A., Glajchen M., Portenoy R.K., Berdella M., Walker P., Barrett M., Chen J., Plachta A., Balzano J., Fresenius A. (2018). Trajectories of caregiver burden in families of adult cystic fibrosis patients. Palliat. Support. Care.

[57] Middour-Oxler B.D.L., Georgiopoulos A., DiFiglia S., Portenoy R., Chaudhary N., Linnemann R. (2022). P246 Improving assessment of palliative care needs among cystic fibrosis children: A Delphi study of the ADAPT-Cystic Fibrosis communication guide. J. Cyst. Fibros..

[58] (2021). Improving Life with CF: A Primary Palliative Care Partnership. MJHS Institute for Innovation in Palliative Care. www.improvinglifewithcf.org.

[59] Fajac I., Daines C., Durieu I., Goralski J.L., Heijerman H., Knoop C., Majoor C., Bruinsma B.G., Moskowitz S., Prieto-Centurion V. (2023). Non-respiratory health-related quality of life in people with cystic fibrosis receiving elexacaftor/tezacaftor/ivacaftor. J. Cyst. Fibros..

[60] Mayer-Hamblett N., Nichols D.P., Odem-Davis K., Riekert K.A., Sawicki G.S., Donaldson S.H., Ratjen F., Konstan M.W., Simon N., Rosenbluth D.B. (2021). Evaluating the Impact of Stopping Chronic Therapies after Modulator Drug Therapy in Cystic Fibrosis: The SIMPLIFY Clinical Trial Study Design. Ann. Am. Thorac. Soc..

[61] Talwalkar J.S., Koff J.L., Lee H.B., Britto C.J., Mulenos A.M., Georgiopoulos A.M. (2017). Cystic Fibrosis Transmembrane Regulator Modulators: Implications for the Management of Depression and Anxiety in Cystic Fibrosis. Psychosomatics.

[62] Baroud E., Chaudhary N., Georgiopoulos A.M. (2023). Management of neuropsychiatric symptoms in adults treated with elexacaftor/tezacaftor/ivacaftor. Pediatr. Pulmonol..

[63] Arslan M., Chalmers S., Rentfrow K., Olson J.M., Dean V., Wylam M.E., Demirel N. (2023). Suicide attempts in adolescents with cystic fibrosis on Elexacaftor/Tezacaftor/Ivacaftor therapy. J. Cyst. Fibros..

[64] Heo S., Young D.C., Safirstein J., Bourque B., Antell M.H., Diloreto S., Rotolo S.M. (2022). Mental status changes during elexacaftor/tezacaftor / ivacaftor therapy. J. Cyst. Fibros..

[65] Zhang L., Albon D., Jones M., Bruschwein H. (2022). Impact of elexacaftor/tezacaftor/ivacaftor on depression and anxiety in cystic fibrosis. Ther. Adv. Respir. Dis..

[66] Spoletini G., Gillgrass L., Pollard K., Shaw N., Williams E., Etherington C., Clifton I.J., Peckham D.G. (2022). Dose adjustments of Elexacaftor/Tezacaftor/Ivacaftor in response to mental health side effects in adults with cystic fibrosis. J. Cyst. Fibros..

